# Use of Allele-Specific FAIRE to Determine Functional Regulatory Polymorphism Using Large-Scale Genotyping Arrays

**DOI:** 10.1371/journal.pgen.1002908

**Published:** 2012-08-16

**Authors:** Andrew J. P. Smith, Philip Howard, Sonia Shah, Per Eriksson, Stefan Stender, Claudia Giambartolomei, Lasse Folkersen, Anne Tybjærg-Hansen, Meena Kumari, Jutta Palmen, Aroon D. Hingorani, Philippa J. Talmud, Steve E. Humphries

**Affiliations:** 1 Centre for Cardiovascular Genetics, British Heart Foundation Laboratories, Institute of Cardiovascular Sciences, University College London, London, United Kingdom; 2 University College London Genetics Institute, Department of Genetics, Environment, and Evolution, University College London, London, United Kingdom; 3 Atherosclerosis Res Unit, Department of Medicine, Karolinska Institutet, Stockholm, Sweden; 4 Department of Clinical Biochemistry, Rigshospitalet, Copenhagen University Hospital and Faculty of Health Sciences, University of Copenhagen, Copenhagen, Denmark; 5 The Copenhagen City Heart Study, Bispebjerg Hospital, Copenhagen University Hospital and Faculty of Health Sciences, University of Copenhagen, Copenhagen, Denmark; 6 Genetic Epidemiology Group, Department of Epidemiology and Public Health, University College London, London, United Kingdom; 7 Centre for Clinical Pharmacology, Department of Medicine, University College London, London, United Kingdom; Stanford University School of Medicine, United States of America

## Abstract

Following the widespread use of genome-wide association studies (GWAS), focus is turning towards identification of causal variants rather than simply genetic markers of diseases and traits. As a step towards a high-throughput method to identify genome-wide, non-coding, functional regulatory variants, we describe the technique of allele-specific FAIRE, utilising large-scale genotyping technology (FAIRE-gen) to determine allelic effects on chromatin accessibility and regulatory potential. FAIRE-gen was explored using lymphoblastoid cells and the 50,000 SNP Illumina CVD BeadChip. The technique identified an allele-specific regulatory polymorphism within *NR1H3* (coding for LXR-α), rs7120118, coinciding with a previously GWAS-identified SNP for HDL-C levels. This finding was confirmed using FAIRE-gen with the 200,000 SNP Illumina Metabochip and verified with the established method of TaqMan allelic discrimination. Examination of this SNP in two prospective Caucasian cohorts comprising 15,000 individuals confirmed the association with HDL-C levels (combined beta = 0.016; *p* = 0.0006), and analysis of gene expression identified an allelic association with LXR-α expression in heart tissue. Using increasingly comprehensive genotyping chips and distinct tissues for examination, FAIRE-gen has the potential to aid the identification of many causal SNPs associated with disease from GWAS.

## Author Summary

The identification of genetic variants associated with complex diseases has rapidly grown through lowering costs of genome sequencing and the use of large-scale genotyping chips based on this sequencing data. There have not been corresponding advances in the identification of causal genetic variants compared to variants simply associated with diseases or traits. Most of these causal variants are thought to be located not within regions coding for proteins, but within genomic regions that regulate the level of protein. We have combined the use of large-scale gene chips with functional analysis, to determine regions of the genome that confer a greater potential for controlling gene regulation dependent on the genotype of that individual. Combining this data with population data and gene expression data, we identify a potential causal variant that alters regulation of LXR-α, a key mediator in lipid metabolism, and show that this variant is associated with HDL-C levels. This methodology provides a model for future analyses to identify further causal variants for disease.

## Introduction

The proliferation of genome-wide association studies (GWAS) has achieved considerable advances concerning the identification of novel genetic loci associated with phenotypic traits and diseases, and also confirmed many established genetic associations. Following GWAS, the next objective in genetics will be identification of the causal variants marked by current GWAS, and determination of the molecular mechanisms altered by these genetic variants. This step will be another major milestone towards realisation of the fundamental goal for GWAS, in developing novel drug targets based on this new genetic information.

Only a small percentage of GWAS hits are themselves non-synonymous coding SNPs, with their expected causality by changing protein structure and function. The majority of GWAS hits occur within intronic and intergenic regions of the genome and are likely to exert their effects at the level of gene regulation [1]. Due to the complex nature of gene regulation [2], with regulatory elements commonly occurring up to 100 kb from a transcription start site (TSS), identifying the causal SNP from potentially hundreds of other SNPs that are simply in near or complete linkage disequilibrium (LD) with one identified from GWAS, is a challenging undertaking.

The ENCODE project has significantly increased our understanding of the location of regulatory elements throughout the genome [3]. Using techniques such as chromatin immunoprecipitation followed by sequencing (ChIP-seq), we now know the genomic binding sites for some of the key transcription factors (TF) involved in gene regulation in a number of experimental tissues. This technique relies on the existence of a ChIP-grade antibody to recognise each DNA-bound transcription factor, and is the major limitation towards the complete characterisation of all human TF binding sites [4]. A more widespread use of ChIP-seq has been the annotation of the genome for histone methylation signatures, such as H3K4me1 and H3K4me3, strong markers of enhancers and promoters [5]. Other sequencing techniques have been used to map the genome for open chromatin, including DNase I hypersensitivity (DNase-seq) [6] and formaldehyde-assisted isolation of regulatory elements (FAIRE-seq) [7]. These regions of open chromatin correlate extremely highly with both histone methylation signatures and TF ChIP-seq, but in contrast to ChIP-seq, are able to identify regulatory regions without prior knowledge of a specific transcription factor involved.

If a non-coding SNP associated with gene regulation were to be functional, it would be expected to alter not only transcription factor binding, but also histone methylation signatures and chromatin accessibility. We have applied this hypothesis to identify the functionality of SNPs on a larger scale than has previously been possible, using gene chip technology. In this paper we describe a method for allele-specific FAIRE using gene chip technology, we term FAIRE-gen, to identify possible candidate functional SNPs in loci related to cardiovascular disease.

## Results

### Use of FAIRE-gen to Determine Allele-Specific Enrichment of Regulatory Regions

**Figure 1:**
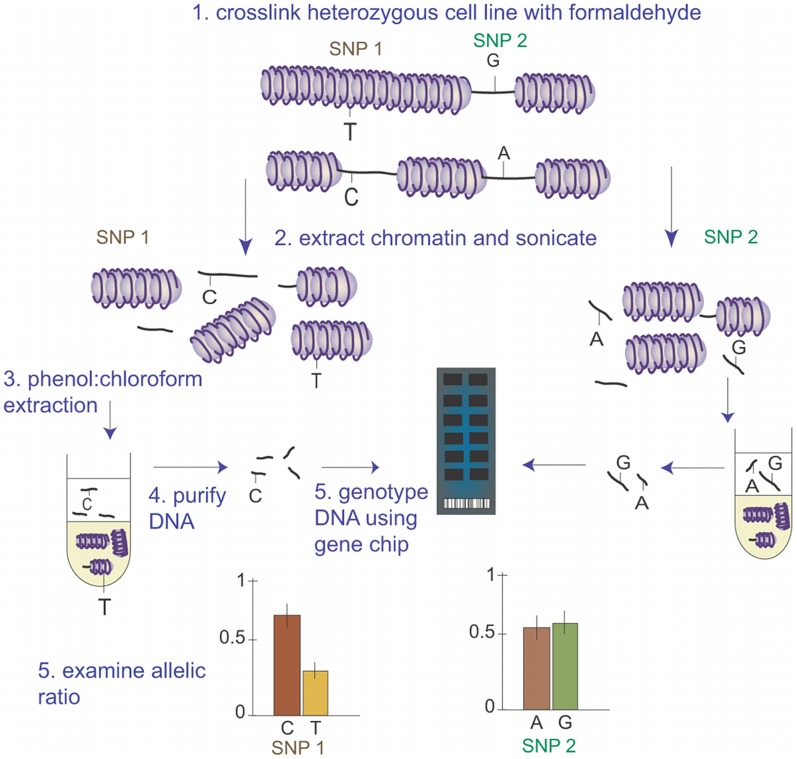
Principle of High-Throughput Analysis of Open Chromatin Using FAIRE-gen. In this example, two potentially functional GWAS SNPs which are in complete LD are illustrated: SNP 1, a T>C, where the C allele occurs in a region of open chromatin, relative to allele T; and SNP 2, a G>A, where both SNPs occur within open chromatin. Following formaldehyde-fixing and sonication, the T allele from SNP 1 remains tightly bound within the nucleosome. Upon phenol:chloroform extraction, this DNA-bound nucleosome transfers to the solvent layer, whilst the C allele within open chromatin remains in the aqueous layer and is purified. Upon genotyping with a gene chip, the C allele is enriched compared to the T allele. For SNP 2, the polymorphism does not affect chromatin structure; both alleles are equally enriched following FAIRE. This would suggest that SNP 1 was the more likely causal SNP for the GWAS association, conferring a greater allele-specific regulatory potential.

To examine the potential to use gene chips to assess allele-specific FAIRE, three lymphoblastoid cell lines were examined following IL-1β stimulation to induce cell proliferation [8]. Subsequent to cell fixing, chromatin extraction and sonication, the fragmented chromatin was divided into two groups for each cell line: a control DNA and a FAIRE-enriched DNA sample. For the control DNA, the crosslinks were reversed and the DNA purified; for the FAIRE-enriched DNA, the chromatin underwent three rounds of phenol:chloroform extraction to enrich the sample for open chromatin, followed by reversal of crosslinks and DNA purification. Both samples were standardised to 50 ng/µl and genotyping performed using the Illumina CVD BeadChip, a custom-designed chip containing 49,094 SNPs from gene loci selected to play a potential role in cardiovascular disease (Figure 1).

**Figure 2:**
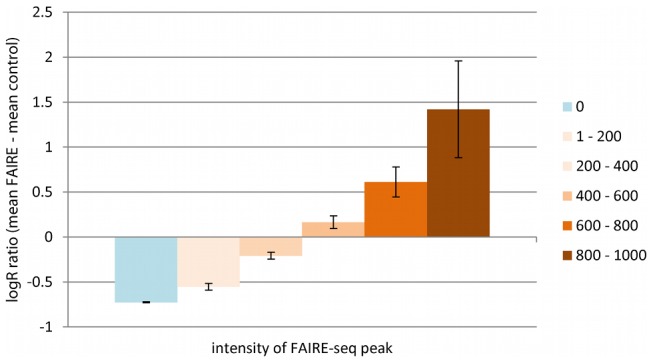
Correlation of FAIRE LogR Ratio with FAIRE-seq Peaks. The graph shows the correlation of FAIRE-enriched SNP intensity using Illumina CVD BeadChip with FAIRE-seq peak intensity from the GM12878 lymphoblast cell line. The (mean log R ratio from three FAIRE-enriched DNAs) – (mean log R ratio from their respective control DNAs) were compared with known FAIRE-seq intensities (0 = no FAIRE-seq enrichment; 1–200 = lowest level of FAIRE-seq enrichment; 800–1000 = highest FAIRE-seq enrichment). There is a strong correlation between SNP intensity and FAIRE-seq peak intensity (p = 2.34×10^−82^).

Genotyping call frequencies for sonicated control DNA were comparable to non-fragmented DNA (97.2% vs 98.1%); whereas those for FAIRE-enriched DNA were significantly lower (56.7%). Using an existing lymphoblastoid FAIRE-seq dataset, the level of enrichment at the location of the CVD BeadChip SNPs was compared with the FAIRE-gen samples. The logR ratio output from the Illumina GenomeStudio was used to indicate the level of allelic amplification and therefore FAIRE-gen enrichment, compared to the respective control samples. A strong association of mean FAIRE-gen-enriched allelic intensity with FAIRE-seq peak intensity was observed (p = 2.34×10^−82^, Figure 2). The reduced amplification of alleles outside of open chromatin results in decreased genotype clustering and a lower call-rate in the FAIRE-enriched samples.

Following FAIRE, an allelic effect on open chromatin would enrich one allele over the other in a heterozygous individual. To examine whether this small dataset was large enough to identify an allele-specific effect on open chromatin, each sonicated control sample and its respective FAIRE-enriched sample was examined using the B allele frequency (BAF), which measures the proportion of the genotype from an individual attributed to the B allele (often the minor allele). To ensure a consistent allelic effect was found, only SNPs that were heterozygous in all three cell lines were examined. This reduced the number of SNPs under analysis to 3,129.

**Figure 3:**
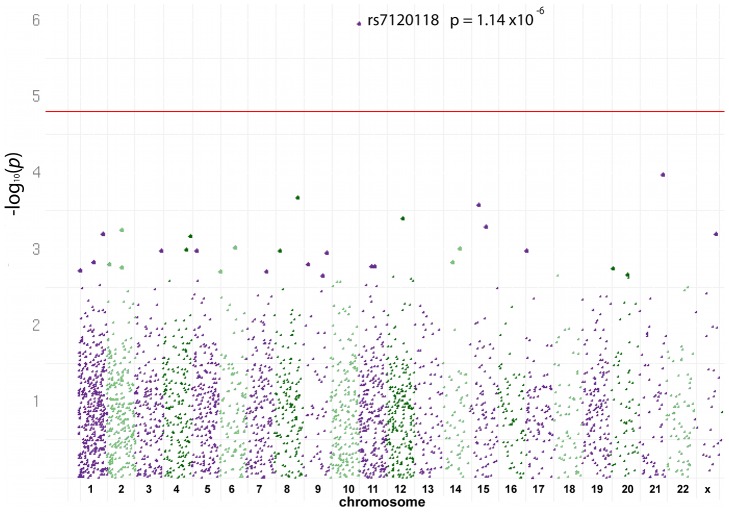
Allele-Specific Open Chromatin Signals from Heterozygous Lymphoblast Cell Lines. Manhattan plot showing allele-specific signals of open chromatin using the Human CVD beadchip. The BAF of chromosome 11 SNP, rs7120118 (C allele), is significantly enriched following FAIRE-gen, in an examination of 3,129 SNP heterozygous SNPs in 3 lymphoblast cell lines. No other SNP showed significant allele-specific effects for open chromatin.

These 3,129 heterozygous SNPs were examined for allelic enrichment, where the control BAF and FAIRE-enriched BAF were compared for each cell line. One SNP showed a statistical significant difference with all three cell lines after applying the Bonferroni correction: rs7120118 (Figure 3), where the C allele was enriched in open chromatin. The fact that only a single association was identified was not unexpected for such a genotyping chip, where the SNP coverage per gene is low and concentrated within coding regions, where the majority of genes covered do not overlap with eQTLs or GWAS studies, and considering the very small number of cell lines examined. Despite only one SNP reaching the Bonferroni cut-off, there was overall enrichment in the study for p-values<0.05 (Figure S1), highlighting the potential for a greater number of significant results with a larger sample.

**Figure 4:**
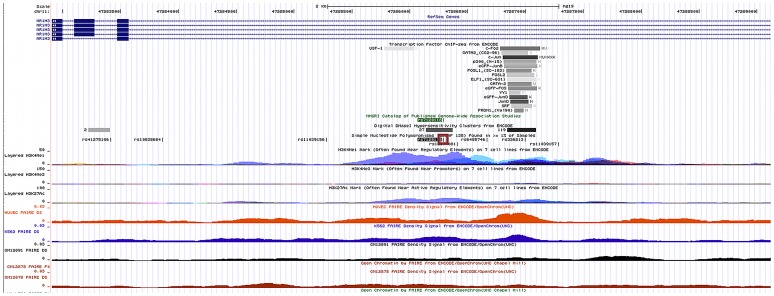
UCSC Genome Browser Annotation of rs7120118 Locus on Human Mar. 2009 (NCBI37/hg19) Assembly. The annotated region surrounding rs7120118 (SNP highlighted in red box) reveals the location of a putative enhancer, with typical features including H3K4me3 signatures, DNase I hypersensitivity and FAIRE-seq enrichment in a number of tissues. The SNP lies between two regions of transcription factor binding sites, including a c-Fos/c-Jun (AP-1 heterodimers), p300 (a transcriptional co-activator), YY1, SRF, GATA-2 complex, and a USF-1 binding site.

The SNP that did show statistical significance is located within intron 6 of *NR1H3*, coding for LXR-α. Examining genomic annotations for this SNP on the UCSC Genome Browser, it can be seen that not only is this SNP located in a region of open chromatin by DNase I-seq [9], [10], FAIRE-seq [7], [11] and with enhancer-specific histone methylation signatures [5], [12] (Figure 4), it has also been identified as a GWAS SNP for HDL-C levels [13].

### Replication of rs7120118 FAIRE-gen Using 200K SNP Illumina Metabochip

To confirm the effects seen using the Illumina CVD BeadChip on rs7120118 with allele-specific FAIRE, the study was replicated using the Illumina Metabochip, a consortia custom-designed genotyping chip, containing 196,726 SNPs to primarily examine associations identified by GWAS for cardiometabolic traits and diseases, those in strong LD, and also a number of rare variants. The Metabochip contains rs7120118, and seven out of the eight further SNPs identified as in complete LD with rs7120118 from the CEU panel in the 1000 Genome Project.

A total of 20 lymphoblastoid cells were examined, including new FAIRE preparations for the original three cell lines. 6 additional cell lines were heterozygous for rs7120118, excluding the three previously examined. Comparing BAF between sonicated controls and FAIRE DNA for these 6 cell lines, the C allele was again enriched in the FAIRE sample (control BAF = 0.44, FAIRE-enriched BAF = 0.67, p = 0.0036).

**Figure 5:**
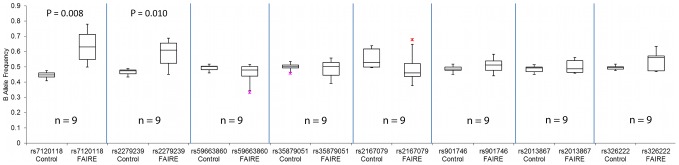
Replication of Allele-Specific Effect of rs7120118 from 9 Heterozygous IL-1β Stimulated Lymphoblast Cell Lines Using Illumina Metabochip. The boxplots indicate the effect size of allele-specific differences in open chromatin. Included are the 7 SNPs in high LD. The B allele (rs7120118 C) is enriched in open chromatin, as is the adjacent SNP, rs2279239 with less statistical significance.

The seven SNPs in complete LD with rs7120118 were examined by the same analysis from the Metabochip using all 9 heterozygous cell lines. Unlike the original Illumina CVD BeadChip assay, Metabochip FAIRE-gen was performed on both unstimulated and IL-1β-stimulated lymphoblastoid cell lines, allowing a direct comparison of IL-1β stimulation on allele-specific open chromatin. The results for all analyses are shown in Table 1. The rs7120118 C allele was enriched with and without IL-1β stimulation by 15.5% (p = 0.008) and 4.4% (p = 0.022), respectively. No other SNPs from the seven in complete LD with rs7120118 in the IL-1β-stimulated cell lines showed allelic enrichment. From the stimulated cell lines there was a trend towards BAF enrichment from the adjacent SNP rs2279239 (11.3%, p = 0.01, Figure 5), contained within the same region of open chromatin, but this did not reach statistical significance when correcting for multiple comparisons. Examining the unstimulated cell lines, two further SNPs showed modest allelic imbalance following FAIRE: rs2167079 (exon 1 of *ACP2*), with a 10.3% reduction in BAF (p = 0.003) and rs326222 (intron 8 of DDB2) with a 4.9% reduction in BAF (p = 0.003).

**Table 1:** Examination of rs7120118 and SNPs in complete LD with this using Metabochip FAIRE-gen in 9 heterozygous lymphoblastoid cell lines.

IL-1β stimulated								
SNP	rs7120118	rs2279239	rs59663860	rs35879051	rs2167079	rs901746	rs2013867	rs326222
control BAF	0.446	0.466	0.494	0.499	0.552	0.485	0.486	0.494
FAIRE BAF	0.601	0.579	0.457	0.491	0.498	0.512	0.505	0.535
difference	0.155	0.113	−0.037	−0.009	−0.055	0.027	0.019	0.040
p	**0.008**	**0.010**	0.111	0.669	0.259	0.192	0.356	0.065

Unstimulated								
SNP	rs7120118	rs2279239	rs59663860	rs35879051	rs2167079	rs901746	rs2013867	rs326222
control BAF	0.469	0.490	0.521	0.498	0.539	0.499	0.503	0.506
FAIRE BAF	0.513	0.514	0.503	0.509	0.436	0.483	0.490	0.458
difference	0.044	0.024	−0.018	0.011	−0.103	−0.016	−0.013	−0.049
p	**0.022**	0.176	0.100	0.447	**0.003**	0.405	0.569	**0.003**

### Use of TaqMan Allelic Discrimination to Confirm Allele-Specific Genotyping

**Figure 6:**
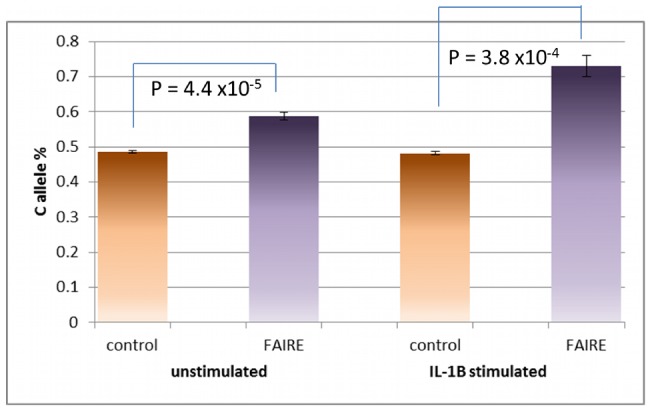
Replication of the Allele-Specific Effect of rs7120118 from 9 Heterozygous Lymphoblast Cell Lines Using the TaqMan Platform. The effect of C allele-enrichment from the Illumina Metabochip is confirmed using an alternative method of allele-quantification from the TaqMan platform.

To confirm the allele-specific enrichment from the C allele of rs7120118, genotyping of the 20 sonicated control and FAIRE samples was carried out using the TaqMan platform for allelic discrimination. Allelic ratios were determined by extrapolation from a standard curve of the vic/fam ratio from samples of known genotype. The allelic ratios do not differ significantly from the Metabochip data, confirming the ability for gene chips to provide a suitable high-throughput method for FAIRE-gen (Figure 6).

### Confirmation of rs7120118 as a Marker for HDL-C Plasma Levels

The SNP showing the greatest and most consistent allelic effect for open chromatin, and confirmed in two subsequent genotyping platforms, rs7120118, has been identified using GWAS as being associated with plasma HDL-C levels [13]. The SNP was associated with a beta coefficient of 0.04 (0.0073 SE, p = 6.7×10^−8^), but this finding has not been replicated in further GWAS, and not reported in a recent meta-analysis of lipid traits comprising >100,000 individuals [14]. To confirm the original association, we examined this SNP in a prospective UK cohort of 4724 individuals from the Whitehall II study. Baseline characteristics of the study are shown in Table 2. This data replicated the reported association with an HDL-C raising effect from the C allele (beta = 0.016, p = 0.0059). No other SNPs in strong LD with this SNP (r^2^>0.5) showed significantly greater effect sizes (Table 3). An additional cohort, the Copenhagen City Heart Study (CCHS; *n* = 10,322, baseline characteristics shown in Table 2) was genotyped for rs7120118, and this also showed a similar effect size (beta = 0.015, p = 0.041, Table 4). Combining the two datasets in a meta-analysis using a fixed-effects model did not alter the effect size (beta = 0.016) although increased the significance (*p* = 0.0006). As there is an association between gender and HDL-C levels in the general population, we also carried out stratification for gender. This showed a similar direction of effect in both studies, showing that the effect seen with rs7120118 functionality is unlikely to be gender-specific. This correlates with the functional *in vivo* findings, where the rs7120118 C allele is associated with open chromatin in cells from both male and female origin (data not shown).

**Table 2:** Baseline characteristics of the Whitehall II study (including all individuals examined on both CVD BeadChip and Metabochip) and the Copenhagen City Heart Study (CCHS).

Baseline characteristics	WHII	CCHS
Total participants, No.	5059	10322
Women (%)	1338 (26)	5754 (56)
Age, years	49 (44–54)	59 (45–69)
Body mass index (kg/m2)	25 (23–27)	25 (22–28)
Total cholesterol (mmol/L)	6.4 (5.7–7.2)	6.0 (5.1–6.9)
HDL cholesterol (mmol/L)	1.4 (1.1–1.7)	1.5 (1.2–1.8)
LDL cholesterol (mmol/L)	4.4 (3.7–5.0)	3.6 (2.9–4.4)
Triglycerides (mmol/L)	1.4 (0.8–1.7)	1.5 (1.1–2.2)

Values are number and (%) or median and (interquartile range).

**Table 3:** Associations of SNPs in LD with rs7120118 and HDL-C levels in 3,413 individuals from the WHII study.

snp	r^2^ with rs7120118	BETA	p
rs10838692	0.959	0.02107	0.002977
rs3816725	0.959	0.02076	0.003413
rs10838681	0.833	0.02038	0.006481
rs11039119	0.544	0.004251	0.5476
rs3758668	0.517	0.01596	0.08267

**Table 4:** Meta-analysis using fixed-effect model of associations of rs7120118 and HDL-C in CCHS and WHII, stratified by gender.

			WHII			CCHS			Meta-Analysis	
SNP	Risk Allele	Samples	Beta (95% CI)	P	N	Beta (95% CI)	P	N	Beta (95% CI)	P
rs7120118	C	all	0.016 (0.0046–0.027)	0.0059	4724	0.015 (0.00063–0.029)	0.041	10322	0.016 (0.0066–0.024)	0.0006
rs7120118	C	males	0.018 (0.0048–0.031)	0.0077	3481	0.088 (−0.00997–0.0276)	0.358	4568	0.015 (0.0041–0.026)	0.0067
rs7120118	C	females	0.0096 (−0.012–0.031)	0.39	1243	0.023 (0.004–0.042)	0.018	5754	0.017 (0.0029–0.032)	0.019

### Effects of rs7120118 on Gene Expression

**Figure 7:**
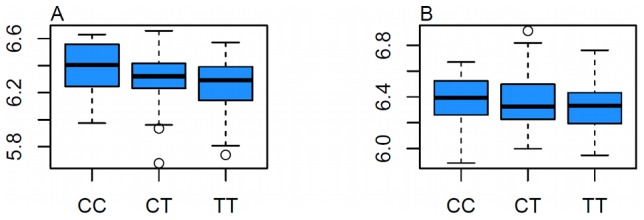
Effect of rs7120118 on *NR1H3* Gene Expression in Tissue. A) Effect of rs7120118 genotype on *NR1H3* gene expression in heart samples (n = 127). The minor allele (C) is associated with increased expression at *p* = 0.0127. B) Effect of rs7120118 genotype on *NR1H3* gene expression in aortic adventitia samples (n = 133). There is a trend towards increased expression of *NR1H3* with the C allele (*p* = 0.154).

To determine if the association of rs7120118 with both HDL-C levels and open chromatin was also associated with an intermediate phenotype of *NR1H3* gene expression, this SNP was examined in five tissue samples from 316 patients undergoing aortic valve surgery. A significant allele-specific effect was observed in heart tissue (p = 0.0127) (Figure 7), with a trend towards significance in aortic adventitia (P = 0.154). In both cases the C allele of rs7120118 was associated with an upregulation of *NR1H3* expression.

## Discussion

**Figure 8:**
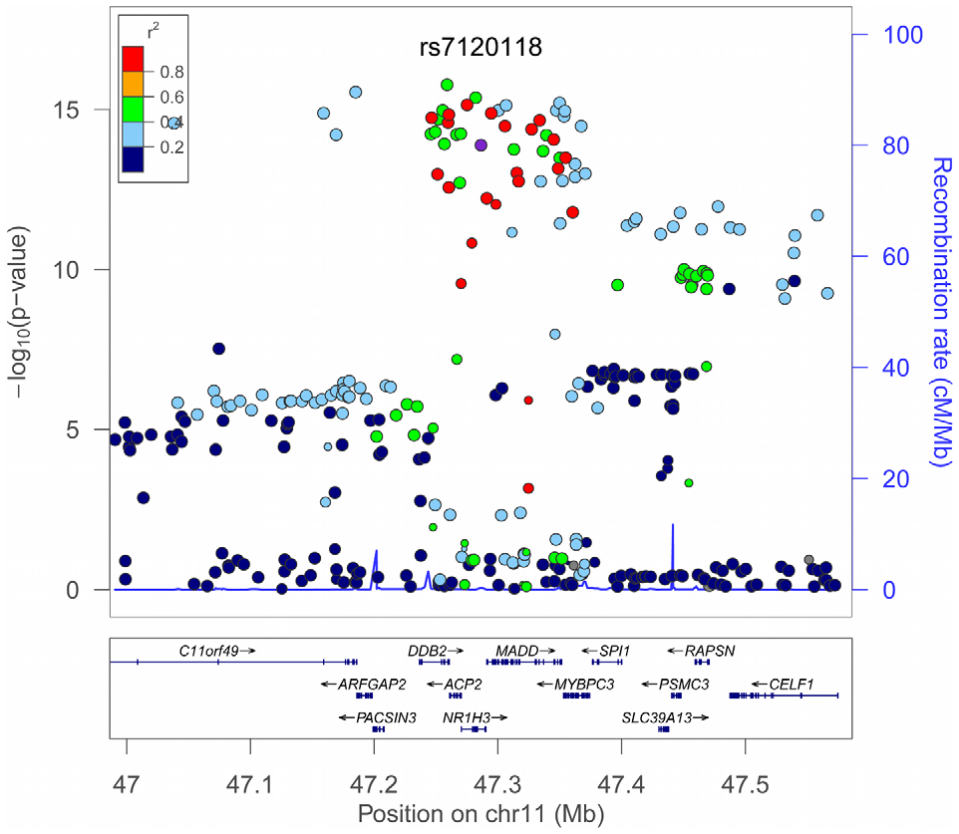
Association of rs7120118 with HDL-C in More Than 100,000 Individuals. Association of rs7120118 with HDL-C levels was examined using a published dataset from a study in more than 100,000 individuals [14] using LocusZoom [27] to plot the SNPs examined and imputed from 1000 Genomes Project dataset. A high level of LD is present within the locus, with at least 29 genes implicated with HDL. rs7120118 is indicated in purple (p = 1.297×10^−14^), with the SNPs in strongest LD marked in red. Although not statistically the lead SNP at this region, with the additional effects of this SNP on open chromatin, *NR1H3* gene expression, and proximity to *NR1H3*, rs7120118 is represents a good functional candidate.

We have examined the possibility of using high-throughput gene chips to examine the allele-specific nature of open chromatin using FAIRE (illustrated in Figure 1). The study identified a functional SNP, rs7120118, where the minor C allele is enriched in open chromatin and associated with increased HDL-C. Although the level of significance for HDL-C levels was adequate for a SNP with an *a priori* hypothesis, this would be much lower than required for genome-wide significance, highlighting the importance of combining functional studies with GWAS to identify candidate SNPs for disease or trait associations, particularly those with lower effect sizes, rare SNPs or small cohorts. Indeed, examining a recent meta-analysis of lipid traits in >100,000 individuals, rs7120118 did show a strong association with HDL-C levels (p = 1.297×10^−14^, Figure 8) although this was not reported as significant in the study [14], perhaps due to the strong LD in the region, with the association signals covering >29 genes. We have shown that the minor allele is associated with increased *NR1H3* gene expression in heart tissue and aortic adventitia, adding to a previous genome-wide study revealing a significant association with rs7120118 and gene expression of *NR1H3* and *ACP2* in lymphoblast cells [15]. From this data it can be postulated that rs7120118 directly affects a long-range regulatory element (>15 kb from *NR1H3* TSS) in a non-tissue-specific manner, altering gene expression and HDL-C levels.

The principle of allele-specific FAIRE was previously applied by Gaulton *et al* to examine the functionality of a single type II diabetes (T2D) GWAS SNP in *TCF7L2* [16]. The authors used FAIRE-seq to determine global tissue-specific regions of open chromatin in pancreatic tissue, followed by TaqMan allelic discrimination to ascertain the effect of a single putative functional SNP on open chromatin. They found that the allele conferring increased risk of T2D and higher gene expression was also associated with enrichment for open chromatin. Although successfully demonstrating the use of FAIRE to identify a causal SNP from a GWAS, the use of TaqMan would not be applicable for examining a large number of potentially functional SNPs. FAIRE-gen, in contrast is only restricted by the number of SNPs that can fit on a genotyping chip.

The action of IL-1β on chromatin structure, a cytokine known to induce proliferation of EBV-transformed lymphoblasts [8], was examined in this study to reveal further potential allele-specific differences in open chromatin under different environmental conditions. For rs7120118, an allele-specific effect was observed in both unstimulated and IL-1β-stimulated cell lines, although the effects were stronger in the IL-1β stimulated samples. The action of IL-1β activates NF-κB, potentially altering expression of transcription factors that bind to the regulatory region surrounding rs7120118. Indeed, a nearby cluster of transcription factor binding sites determined by ChIP-seq includes a site for c-Jun binding (Figure 4); the *JUN* promoter contains several NF-κB binding sites (UCSC Genome Browser hg19/NCBI37) [3], which may explain this enhanced effect. It could be hypothesised that the C allele that favours open chromatin allows for preferential access for known, or as yet uncharacterised, transcription factors, which would act as an enhancer for *NR1H3* gene expression, and increased HDL-C levels.

In contrast, a potential allelic effect was observed with the promoter SNP rs2167079 (in complete LD with rs7120118), only in unstimulated cells. IL-1β is known to reduce expression of *NR1H3* in HK-2 cells [17], and it could be postulated that IL-1β may lead to chromatin remodelling and a decrease in open chromatin at the *NR1H3* promoter in lymphoblasts, accounting for the lack of allelic effect in the IL-1β-stimulated cells. Alternatively, the modest allele-specific chromatin effects from the unstimulated cell lines could simply represent false-positive findings.

Haplotype structure may also affect local chromatin, particularly where more than one SNP occurs in the same region of open chromatin. We have examined the variation surrounding rs7120118 using HapMap-derived genotypes for the lymphoblasts used in the Metabochip study. No further SNPs at the locus provided additional haplotypic information for the effects on open chromatin, suggesting that rs7120118, rather than a haplotype, is responsible for this observation.

To assess the reproducibility of the FAIRE-gen methodology, the two Metabochip datasets were examined, considering the second IL-1β-treated study as a replicate. Examining the SNPs showing an allele-specific effect on open chromatin from the untreated samples, following Bonferroni correction (p<5.2×10^7^; *n* = 127), 100% were replicated in the treated sample with significance set at p<0.05, (91% replicated with *P_c_*<3.9×10^−4^; *n* = 116), indicating the sensitivity of the assay. The sensitivity and specificity of the assay to identify true functional variants can only be accurately determined by further functional analysis of each putative SNP. The smallest detectable difference in allele-specific open chromatin for the SNPs reaching genome-wide Bonferroni cut-off in the Metabochip was 10% (rs75106522).

One limitation with FAIRE-gen, as opposed to FAIRE-seq is the dependence of the gene chip to contain all relevant SNPs for the trait under examination. For the recent custom-designed chips which contain dense markers and aim to include all SNPs that tag GWAS-identified markers for diseases and related traits, such as the Illumina Metabochip and Immunochip, this is less of a problem. Future genotyping chips containing all common SNPs associated with diseases/traits could potentially resolve this drawback. For determining the location of potential causal SNPs from a number of SNPs acting as proxies, FAIRE-gen is only able to identify single allele-specific SNPs if other proxies are not located within the same region of open chromatin. This can be illustrated for rs7120118, where a nearby SNP, rs2279239, is located only 4.6 kb away, and close to the same region of open chromatin (Figure S2). This SNP shows a similar trend for allelic-specificity, although somewhat reduced due to the distance from the putative causal SNP.

Since the assay includes data from SNPs that are not present in open chromatin, there may also be a number of false-positive associations from the methodology, where amplification from background (non-open) chromatin may, in theory, preferentially occur for one allele. For this reason, replication using FAIRE-gen or FAIRE-seq in a separate study, and *in vitro* methodologies would be desirable to confirm functionality.

In conclusion, FAIRE-gen shows promise as an economical, high-throughput method to enable targeted unbiased detection of allele-specific regulatory elements, which may help to refine GWAS disease-association signals to identify disease-causing variants.

## Materials and Methods

### Ethics Statement

The Whitehall II study was approved by the UCL Research Ethics Committee, and participants gave informed consent to each aspect of the study. The CCHS was approved by institutional review boards and Danish ethical committees, and conducted according to the Declaration of Helsinki. Written informed consent was obtained from all participants.

### Cell Lines and Culture

20 EBV-transformed lymphoblastoid cell lines, derived from the Centre d'Etude du Polymorphism Humain (CEPH) panel (Coriell Cell Repositories, identifiers listed in table S1), were cultured in RPMI 1640 (PAA) with 2 mM L-glutamine and 15% fetal bovine serum (PAA) at 37°C, 5% CO_2_. Cell viability was verified using the ADAM-MC cell counter (Digital Bio), and minimum cell viability for experiments was ≥99%. Stimulation of cells was carried out by an overnight incubation in serum-free media, and addition of 5 ng/ml IL-1β, two hours prior to cell fixing.

### Chromatin Fixing, Isolation, and Sonication

1×10^8^ cells were cultured for each experiment and incubated with 1/10 volume of fresh 11% formaldehyde for 20 min. 1/20 volume of 2.5 M glycine was added to quench formaldehyde. Cells were washed 3 times in PBS and resuspended in 10 ml lysis buffer 1 (50 mM HEPES-KOH, pH 7.5, 140 mM NaCl, 1 mM EDTA, 10% glycerol, 0.5% NP-40, 0.25% Triton-X-100, 1× protease inhibitors) for 10 min. After centrifugation, the supernatant was discarded and pellet resuspended in 10 ml lysis buffer 2 (10 mM Tris-HCL, pH 8.0, 200 mM NaCl, 1 mM EDTA, 0.5 mM EGTA, 1× protease inhibitors) for 10 mins. The nuclei were pelleted and resuspended in 3.5 ml lysis buffer 3 (10 mM Tris-HCL, pH 8.0, 100 mM NaCl, 1 mM EDTA, 0.5 mM EGTA, 0.1% NA Deoxycholate, 0.5% *N*-lauroylsarcosine, 1× protease inhibitors). Sonication was performed using the Bioruptor sonicator (Wolflabs, York, UK) and optimized to produce maximum enrichment of fragments 100–1000 bp, prior to downstream analysis. 1/10 volume of 10% Triton X was added to the sonicated sample, the sample centrifuged at 20,000 g and the lysate stored on ice.

### Formaldehyde-Assisted Isolation of Regulatory Elements (FAIRE) and Genotyping

Following chromatin fixing, isolation and sonication, the sheared lysate was subject to three rounds of phenol:chloroform extraction, followed by a final chloroform extraction. The DNA was ethanol precipitated and the pellet resuspended in TE buffer. The DNA solution was treated with 0.2 mg/ml RNase A and incubated at 37°C, and 0.2 mg/ml proteinase K at 55°C for two hours. Samples were incubated at 65°C overnight to remove crosslinks. The samples were subjected to a further phenol:chloroform extraction and ethanol precipitation and standardised to 50 ng/ml for Illumina genotyping chips. For each respective control sample, 10% of the fixed and sonicated chromatin was reverse-crosslinked at 65°C overnight, treated with 0.2 mg/ml RNase A and incubated at 37°C for two hours and 0.2 mg/ml proteinase K at 55°C for 2 hours. The samples underwent 3 rounds of phenol:chlororom extraction followed by ethanol precipitation and standardisation to 50 ng/ml for Illumina genotyping. Genotyping was carried out using the Illumina CVD BeadChip and Illumina Metabochip. Genotype calls for control samples were generated using Illumina GenomeStudio software. Call rates for control and FAIRE samples are described in the Results.

### Whitehall II Study (WHII)

DNA was extracted from whole blood. Genotyping for 6,156 samples and laboratory analysis of has been described previously [18]. 5529 samples were genotyped using the Illumina CVD BeadChip [19] and 3,413 samples were genotyped using the Illumina Metabochip. Genotype calls were generated using Illumina GenomeStudio software. After filtering for duplicates, cryptic relatedness, ambiguous gender, self-reported non-Caucasians, outliers based on the genome-wide identity-by-state analysis implemented in PLINK, sample call rate>80% and SNP call rate>98%, 5059 CVD BeadChip and 3126 Metabochip genotyped samples were available for analysis.

### Copenhagen City Heart Study (CCHS)

The CCHS [20], [21] is a prospective study of the Danish general population initiated in 1976–78 with follow-up examinations in 1981–84, 1991–94, and 2001–03. Individuals were randomly selected to represent the Danish general population aged 20 to 80+ years. We included 10,322 participants who gave blood for DNA analysis at the 1991–94 and/or 2001–03 examinations. The study was approved by institutional review boards and Danish ethical committees, and conducted according to the Declaration of Helsinki. Written informed consent was obtained from all participants. Plasma levels of total cholesterol, LDL cholesterol, HDL cholesterol, and triglycerides were measured using standard hospital assays (Konelab, Helsinki, Finland, and Boehringer Mannheim, Mannheim, Germany). LDL cholesterol was calculated using the Friedewald equation if the triglyceride level was less than 4 mmol per liter (354 mg per deciliter) and was measured directly for higher triglyceride levels. Follow-up studies of rs7120118 in the samples from Copenhagen were performed using an ABI PRISM 7900HT Sequence Detection System (Applied Biosystems Inc, Foster City, California, USA) and a TaqMan-based assay.

### Expression Studies

Tissue biopsies (mammary artery, ascending thoracic aorta and liver) were taken from patients undergoing aortic valve surgery as part of the Advanced Study of Aortic Pathology (ASAP) study [22]. Aortic biopsies were divided into intimal-medial and adventitial halves. Peri-aortic fat was removed from the adventitial specimens where present. RNA from the tissue biopsies was hybridized to Affymetrix ST 1.0 Exon arrays and obtained scans were RMA normalized and log2 transformed. eQTL analysis was performed with an imputed genotype from circulating blood DNA (Illumina 610w-Quad BeadArrays). The full methods for this study have been described previously [22].

### Statistical Analysis

Comparison of the GM12878 lymphoblast FAIRE-seq data track was obtained from the UCSC Genome Browser (http://hgdownload.cse.ucsc.edu/goldenPath/hg18/encodeDCC/wgEncodeChromatinMap/wgEncodeUncFAIREseqZinbaGm12878.narrowPeak.gz) and compared to (mean log R ratio of SNPs following FAIRE-enrichment) - (mean log R ratio for the respective control SNPs). The mean SNP log R ratios stratified by strength of FAIRE-seq signal were compared by ANOVA. A paired two-sided t-test was used to compare the control BAF with the respective FAIRE-enriched BAF. Visualisation of Manhattan plots and data management from the UCSC Genome Browser was carried out using Galaxy software [23]–[25]. In WHII, linear regression analysis of log-transformed HDL-C with SNPs using an additive model was performed using PLINK 1.0.7. Analysis was carried out in all individuals and stratified by gender. Regression analysis was performed unadjusted for covariates as well as gender (only in analysis of all individuals) and age added as covariates. Stata software, version 10 (Stata Corp, College Station, Texas) was used for all analyses in the CCHS. Trend tests were by Cuzick's nonparametric test for trend. Linear regression was used to determine per-allele β-coefficients. For trend tests and linear regression analysis, rs7120118 TT, TC and CC genotypes were coded as 0, 1, and 2, respectively. Statistical analysis of gene expression was carried out using R-2.13.0 and Bioconductor 2.8 [26]. Association between gene expression and genotype was calculated using an additive linear model as implemented in the *lm*-function in R.

## Supporting Information

Figure S1 Histogram of FAIRE-gen p-values for 50K CVD BeadChip. The use of FAIRE-gen on the CVD BeadChip was carried out with a very small number of samples, resulting in only one SNP showing chip-wide significance in relation to chromatin structure. The enrichment of p-values<0.05, indicates the potential for a greater level of functionality to be derived from the genotyping chip with the use of increased sample numbers.
(TIF)

Figure S2 UCSC Genome Browser Chromatin Annotations for Variants in Complete LD with rs7120118. The map shows the location of 8 SNPs in complete LD with rs7120118. Lymphoblast open chromatin and H3K4me1 marks derived from the UCSC Genome Browser are annotated. The regions of distinct enhancers are highlighted in red, illustrating the location of SNPs in complete LD with rs7120118 are in separate regions of open chromatin to this SNP. The association of rs7120118 with open chromatin is unlikely to be marking effects on open chromatin from other SNPs in LD, although the nearest SNP in complete LD (rs2279239) shows a similar, albeit reduced, effect from FAIRE.
(TIF)

Table S1 Lymphobast cell lines used for FAIRE-gen. The cell lines used for the CVD BeadChip and the Metabochip study are indicated.
(DOCX)
