# Supplementary figures and images for: Use of Allele-Specific FAIRE to Determine Functional Regulatory Polymorphism Using Large-Scale Genotyping Arrays
Source: PLoS Genet. 2012 Aug 16;8(8):e1002908. doi: 10.1371/journal.pgen.1002908 (PMC3420950; doi:10.1371/journal.pgen.1002908)

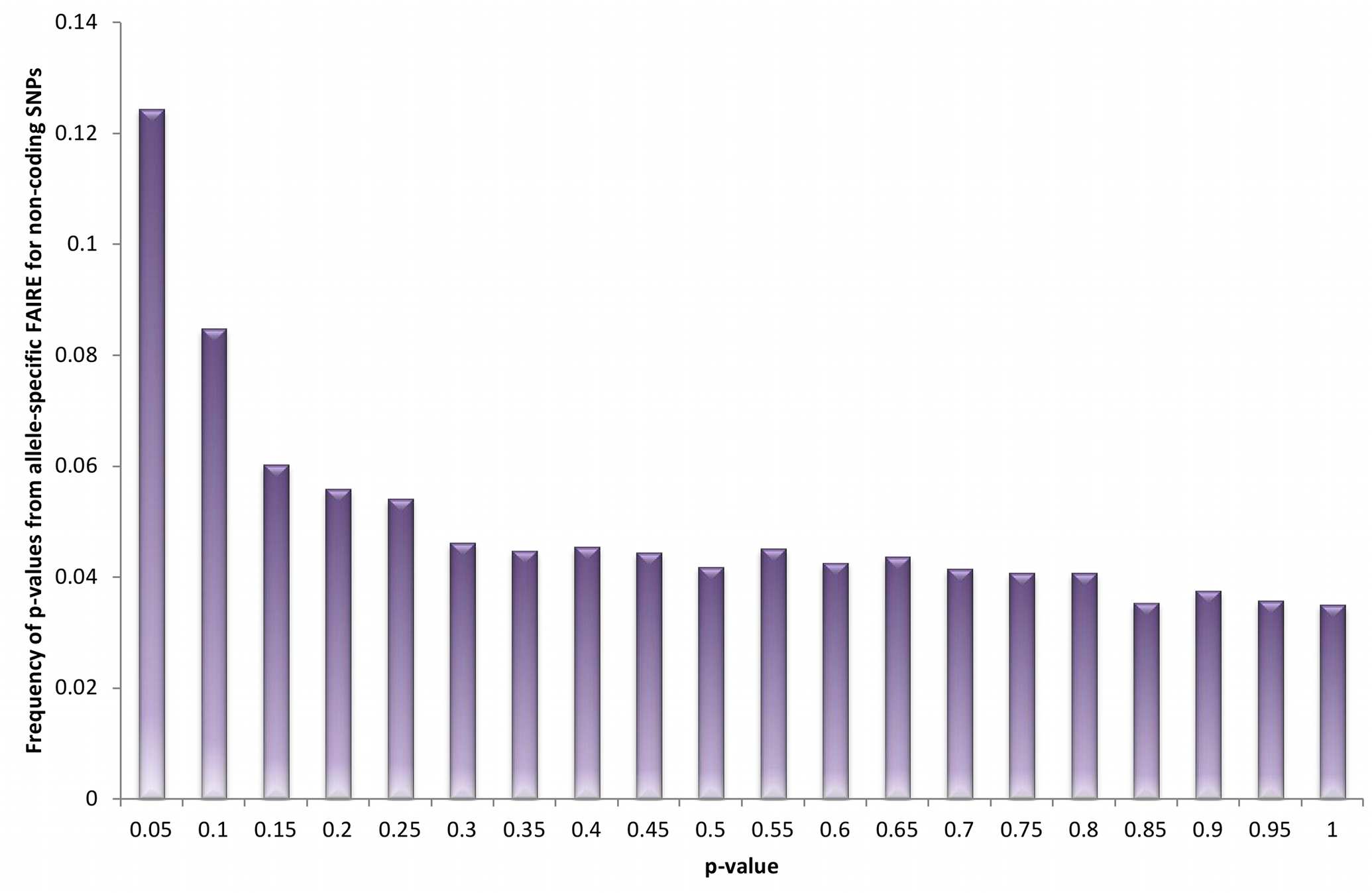

Supplement: Figure S1 — Histogram of FAIRE-gen p-values for 50K CVD BeadChip. The use of FAIRE-gen on the CVD BeadChip was carried out with a very small number of samples, resulting in only one SNP showing chip-wide significance in relation to chromatin structure. The enrichment of p-values<0.05, indicates the potential for a greater level of functionality to be derived from the genotyping chip with the use of increased sample numbers. (TIF) [file pgen.1002908.s001.tif]

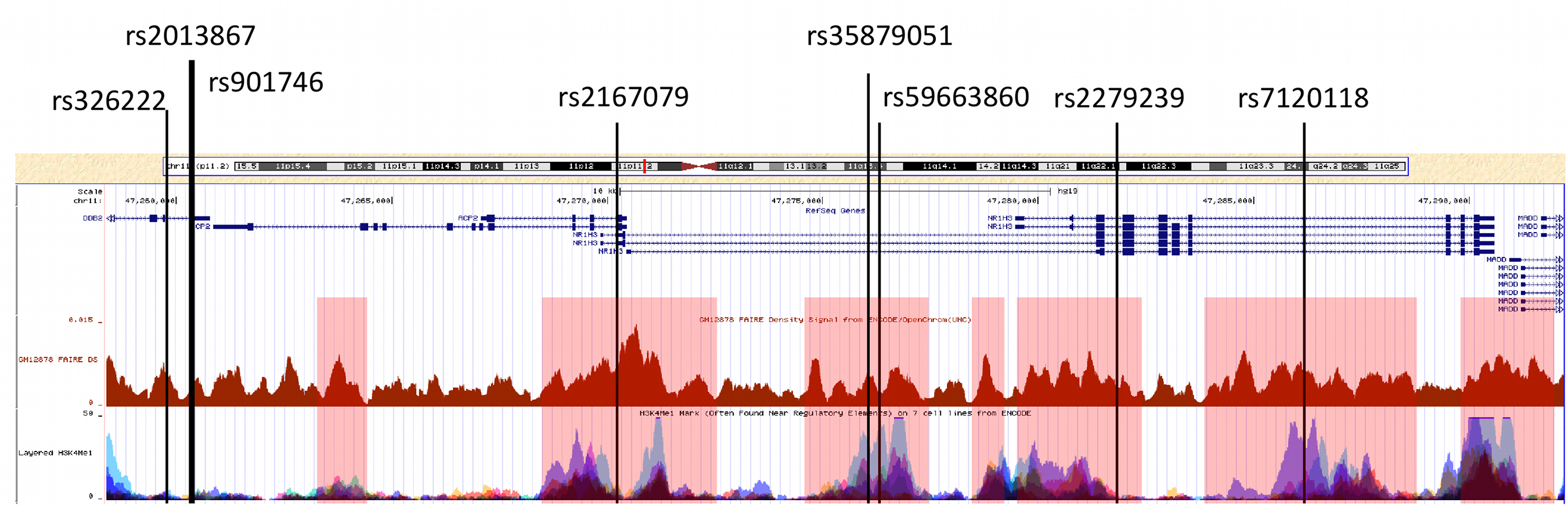

Supplement: Figure S2 — UCSC Genome Browser Chromatin Annotations for Variants in Complete LD with rs7120118. The map shows the location of 8 SNPs in complete LD with rs7120118. Lymphoblast open chromatin and H3K4me1 marks derived from the UCSC Genome Browser are annotated. The regions of distinct enhancers are highlighted in red, illustrating the location of SNPs in complete LD with rs7120118 are in separate regions of open chromatin to this SNP. The association of rs7120118 with open chromatin is unlikely to be marking effects on open chromatin from other SNPs in LD, although the nearest SNP in complete LD (rs2279239) shows a similar, albeit reduced, effect from FAIRE. (TIF) [file pgen.1002908.s002.tif]
