# Supplementary material for: Use of Allele-Specific FAIRE to Determine Functional Regulatory Polymorphism Using Large-Scale Genotyping Arrays
Source: PLoS Genet. 2012 Aug 16;8(8):e1002908. doi: 10.1371/journal.pgen.1002908 (PMC3420950; doi:10.1371/journal.pgen.1002908)
Supplement: Table S1 — Lymphobast cell lines used for FAIRE-gen. The cell lines used for the CVD BeadChip and the Metabochip study are indicated. (DOCX) [file pgen.1002908.s003.docx]

| GM06991 | CVD BeadChip +Metabochip |
| --- | --- |
| GM12865 | CVD BeadChip +Metabochip |
| GM07056 | CVD BeadChip +Metabochip |
| GM12004 | Metabochip |
| GM07000 | Metabochip |
| GM11995 | Metabochip |
| GM11829 | Metabochip |
| GM10830 | Metabochip |
| GM12145 | Metabochip |
| GM10864 | Metabochip |
| GM07055 | Metabochip |
| GM07022 | Metabochip |
| GM06994 | Metabochip |
| GM12155 | Metabochip |
| GM07345 | Metabochip |
| GM07029 | Metabochip |
| GM10831 | Metabochip |
| GM06993 | Metabochip |
| GM07346 | Metabochip |
| GM11839 | Metabochip |

Table S1

Lymphobast cell lines used for FAIRE-gen
